# The Interactive Account of ventral occipitotemporal contributions to reading

**DOI:** 10.1016/j.tics.2011.04.001

**Published:** 2011-06

**Authors:** Cathy J. Price, Joseph T. Devlin

**Affiliations:** 1 Wellcome Trust Centre for Neuro-imaging, University College London, London WC1N 3BG, UK; 2 Cognitive, Perceptual and Brain Sciences, Division of Psychology and Language Sciences, University of London, London WC1E 6BT, UK

## Abstract

The ventral occipitotemporal cortex (vOT) is involved in the perception of visually presented objects and written words. The Interactive Account of vOT function is based on the premise that perception involves the synthesis of bottom-up sensory input with top-down predictions that are generated automatically from prior experience. We propose that vOT integrates visuospatial features abstracted from sensory inputs with higher level associations such as speech sounds, actions and meanings. In this context, specialization for orthography emerges from regional interactions without assuming that vOT is selectively tuned to orthographic features. We discuss how the Interactive Account explains left vOT responses during normal reading and developmental dyslexia; and how it accounts for the behavioural consequences of left vOT damage.

## The diverse response properties of vOT

There has been considerable interest in the role of the ventral occipitotemporal cortex (vOT) during reading. Learning to read increases left vOT activation in response to written words [1,2] and damage to left vOT impairs the ability to read [3–6]. These and other findings have led to claims that the response properties of vOT change during reading acquisition, leading to neuronal populations that are selectively tuned to orthographic inputs [7,8]. However, a significant number of studies have reported that, even after learning to read, vOT is highly responsive to non-orthographic stimuli, with a selectivity that depends on the nature of the task and the stimulus [9–11]. The same vOT area also responds to orthographic and non-orthographic tactile stimuli [12–15]. These diverse response properties suggest that vOT contributes to many different functions that change as it interacts with different areas [1,9,11,15–21]. In this context, it is difficult to find a functional label that explains all vOT responses.

To explain the heterogeneity of responses in vOT, we formalize the Interactive Account of vOT function during reading by presenting it within a predictive coding (i.e. a generative) framework [22,23]. This perspective provides a parsimonious explanation of empirical findings and is based on established theoretical and neurobiological principles. Before presenting this framework, we begin with an anatomical description of vOT.

## The anatomy of vOT

**Figure 1:**
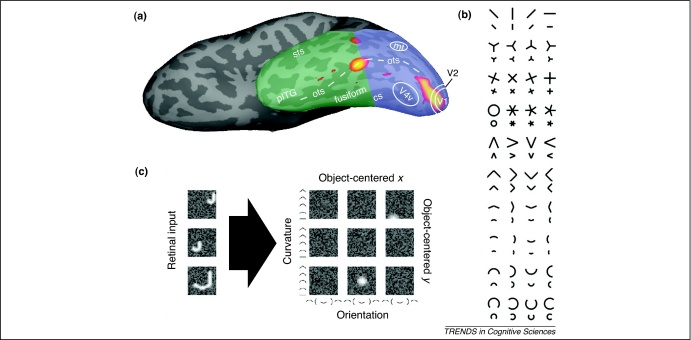
Visual word recognition in the ventral occipitotemporal cortex (vOT). **(a)** The anatomy of vOT and its relation to activation for visual word recognition (red-yellow) shown on the ventral surface of an inflated left hemisphere. vOT is centred on the occipitotemporal sulcus (broken white line) at the transition from the occipital (blue) to the temporal lobe (green).**(b)** Examples of simple shape stimuli that are important for recognizing both visual words and objects. Neurons within V2 respond to these types of simple shapes and project to V4, where the cells have more complex receptive fields that respond to combinations of these shapes within a retinotopic reference frame. These in turn project to vOT neurons that have receptive fields with multidimensional tuning functions, where simple shape elements are combined nonlinearly in an object-centred reference frame. Thus, unlike earlier visual areas, it is difficult – if not impossible – to find the optimal stimulus driving a cell using a simple line drawing. Adapted with permission from [51]. **(c)** A hypothetical example of a complex, object-centred receptive field for a vOT neuron. On the left are three ‘J's of different sizes in different retinal positions. Within early retinotopic areas, each J would be encoded by non-overlapping sets of neurons. By contrast, the receptive field illustrated on the right by a three by three grid of panels provides a more compact, stable object-centred representation. Here, curvature and orientation are plotted recursively within each receptive field region such that it will respond strongly to any combination of a vertical straight line at the top right and a concave-up curved horizontal line at the bottom. Although it is tempting to call this a ‘J-detector’, this would be incorrect – the receptive field responds equally well to the handle of an umbrella or trunk of an elephant but does not respond to the letter j written in script. Reproduced with permission from [52]. cs, collateral sulcus; mt, visual motion area; ots, occipitotemporal sulcus; pITG, posterior inferior temporal gyrus; sts, superior temporal sulcus; V1, central field of primary visual cortex; V2, secondary visual cortex; V4v, ventral component of visual area 4.

vOT is centred on the occipitotemporal sulcus but extends medially onto the lateral crest of the fusiform gyrus and laterally onto the medial crest of the inferior temporal gyrus. In the posterior–anterior direction, vOT is located on the ventral border of the occipital and temporal lobes (Figure 1a), which lies between *y* = –50 and *y* = –60 in standard Montreal Neurological Institute (MNI) space. More posteriorly, activation is highest to visual inputs, but more anteriorly activity increases in response to familiar visual, tactile or auditory stimuli [24], consistent with a basal temporal language area [25]. Given its position between visual and language areas, it is not surprising that vOT responds to a range of visual stimuli as well as the language demands of the task [1,9,11,15–21]. The association between vOT and language processing is further supported by observations that lateralization (left versus right hemisphere dominance) in vOT correlates with language lateralization in frontal language areas [26].

## The Interactive Account of vOT function

**Figure 2:**
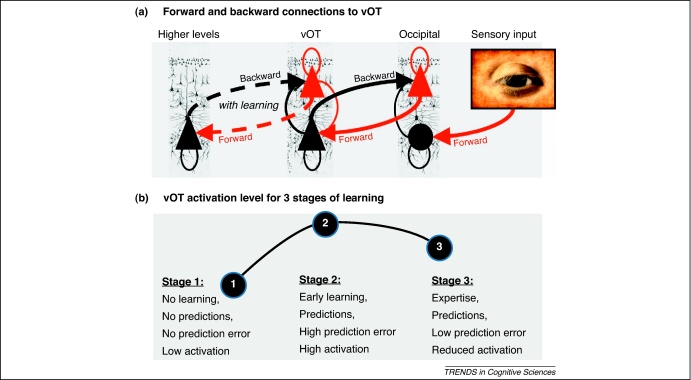
Activation in ventral occipitotemporal cortex (vOT) according to the predictive coding framework. The schematic in **(a)**, adapted from [22], outlines the hierarchical architecture that underlies neuronal responses involved in the perception of visual inputs according to the predictive coding framework [22]. It shows the putative (pyramidal) cells that send forward driving connections (red) from the supragranular cortical layer; and nonlinear (modulatory) backward connections (black) from the infragranular layer. The backward connections predict the response to the forward connections. Predictions are optimized to minimize prediction error at each level in the hierarchy. Prediction error is the difference between the top-down prediction and the representations being predicted at each level. Prediction errors change the predictions through recurrent neuronal message passing until the error is minimized. Recurrent connectivity between different levels of the hierarchy is optimized by experience and therefore depends on learning (as illustrated by the broken lines between vOT and higher levels). In functional magnetic resonance imaging, activation is a measure of combined neuronal firing from the stimulus, predictions and their prediction error.**(b)** Inverted-U shape of activation levels in vOT across three stages of learning. Before learning (stage 1), activation from top-down predictions is precluded because stimuli cannot elicit them (because the appropriate associations have not been learned). This would be the case, for example, in pre-literates and illiterates viewing orthographic stimuli that have no semantic or phonological associations [53] or in literates viewing an unknown orthography (e.g. English readers viewing Chinese characters or an artificial orthography) [1]. In contrast, vOT activation levels are highest during learning (stage 2), when the stimulus is recognized as potentially meaningful (with semantic or phonological associations) but is not predicted efficiently (high prediction error). An example here would be when subjects view pseudowords (that engage high-level representations) but cannot predict their visual form efficiently [41]. With practice, exposure and experience-dependent learning or expertise (stage 3), prediction error decreases and vOT activation declines. The difference between stages 2 and 3 explains why vOT responses are lower for high versus low frequency words [43], real words relative to pseudowords [42] and when words are primed by identical words versus pseudowords [45].

The Interactive Account is based on the premise that perception involves recurrent or reciprocal interactions between sensory cortices and higher order processing regions via a hierarchy of forward and backward connections (Figure 2) [22]. Within the hierarchy, the function of a region depends on its synthesis of bottom-up sensory inputs conveyed by forward connections and top-down predictions mediated by backward connections. These predictions are based on prior experience and are needed to resolve uncertainty and ambiguity about the causes of the sensory inputs on which predictions are based. The hierarchical nature of neocortical organization is reflected in the abundance of backward relative to forward connections [27]. Because functional magnetic resonance imaging (fMRI) does not distinguish between synaptic activity induced by forward connections and that induced by backward connections, it reports their combined contribution (Figure 2), which includes prediction error.

For reading, the sensory inputs are written words (or Braille in the tactile modality) and the predictions are based on prior association of visual or tactile inputs with phonology and semantics. In cognitive terms, vOT is therefore an interface between bottom-up sensory inputs and top-down predictions that call on non-visual stimulus attributes. Without prior knowledge the relationship between orthography and phonology, vOT activation to words will be low because phonological areas do not send backward predictions to vOT (Figure 2 and Box 1). Once phonological associations are learned, backward connections can deliver top-down predictions to vOT when the stimuli are words or word-like. In this context, top-down processing does not imply a conscious strategy; it is mandated by unconscious (hierarchical) perceptual inference. In other words, it represents the intimate association between visual inputs and higher level linguistic representations that occurs automatically and is modulated by attention and task demands. Interpreting activation in vOT therefore requires consideration of the stimulus, experience-dependent learning and context (i.e. the task requirements and the attentional demands). Likewise, interpreting the effect of damage to vOT depends on how word recognition is affected by disrupting top-down inputs from higher order regions to vOT, and from vOT to lower level visual regions (Box 2).

Our account assumes that neuronal populations in vOT are not tuned selectively to orthographic inputs (Box 3). Instead, orthographic representations emerge from the interaction of backward and forward influences. In the forward direction, we postulate that neurons in vOT accumulate information about the elemental form of stimuli from complex receptive fields (Figure 1 and Box 3). In the backward direction, higher order conceptual and phonological knowledge predicts the pattern of activity distributed across multiple neurons within vOT. Put another way, orthographic representations are maintained by the consensual integration of visual inputs with higher level language representations [17,19,20]. This perspective allows the same neuronal populations to contribute to different functions depending on the regions with which they interact and the predictions for which the current context calls. In this context, the neural implementation of classical cognitive functions (e.g. orthography, semantics, phonology) is in distributed patterns of activity across hierarchical levels that are not fully dissociable from one another.

The visual information that is accumulated in vOT must be sufficiently specific to induce coherent patterns of activation in semantic and phonological areas that send top-down predictions back to vOT. For example, in McClelland and Rumelhart's [28] Interactive Activation model of visual word recognition, partial visual information cascades forward activating incomplete phonological and semantic patterns, which in turn feed back to support consistent orthographic patterns and suppress inconsistent ones. As in connectionist models of reading [29–31], we propose that patterns of activation across vOT neurons encoding shape information are sufficient to partially activate neurons encoding semantics and phonology in higher order association regions, which provide recurrent inputs to vOT until the top-down predictions and bottom-up inputs are maximally consistent. Thus, predictions are optimized during the synthesis of bottom-up and top-down information (Figure 2).

## Evidence for automatic (non-strategic) top-down influences on vOT

In cognitive terms, top-down processing typically refers to conscious, strategic and task-related effects. Automatic, non-strategic top-down processes are also recognized, particularly in computational models of reading [23,28,31–33]. The ubiquity of automatic top-down effects has been demonstrated neurophysiologically in monkeys, where inactivating higher-order cortical areas (by cooling) results in changes to extra-classical receptive fields, despite the monkey being anesthetized [34,35].

Here we make a clear distinction between strategic and non-strategic top-down influences on vOT activation. Strategic influences have been demonstrated in studies showing that vOT activation changes with task, even when the stimulus, attention and response times are controlled [9,21,36,37]. In contrast, non-strategic top-down influences on vOT activation are generated automatically and unconsciously from previous experience with similar stimuli (Figure 2 and Box 1). That is, visual words automatically engage processing of their sounds and meaning, which provide predictive feedback to the bottom-up processing of visual attributes.

A clear example of automatic (non-strategic) top-down effects on vOT activation comes from a picture-word priming experiment that found reduced vOT activation for unconsciously perceived primes that were conceptually and phonologically identical to a stimulus that was subsequently named [38]. For example, when a visually presented written object name (e.g. LION) was preceded by a rapidly presented, masked (unconscious) picture of the same object, activation in vOT was reduced relative to when it was preceded by a picture of a different object (e.g. a chair). Similarly, masked written object names (words) reduced vOT activation for pictures of the same objects. These findings can be explained easily by automatic, top-down predictions that prime visual shape information in vOT. In essence, the brief (and unconsciously perceived) prime is sufficient to engage phonological and/or semantic processing that automatically sends predictions regarding the identity of the next stimulus (the target) back to vOT, thereby reducing prediction error and activation. The fact that priming occurs across stimulus formats (pictures/words) demonstrates that these backward projections predict all visual forms of a concept (e.g. object form and written form). The same account also explains reduced vOT activation when a word is primed by the same word in a different case (e.g. AGE–age) without postulating the need for abstract visual word form detectors [17,39].

The effect of word–picture priming on vOT activation cannot be explained in terms of feed-forward visual processing because there is no visual similarity between the prime and the target that can serve as the basis for reduced vOT activation (e.g. through simple adaptation effects). Explanations based on strategic top-down processing are also insufficient, because participants are not aware of the primes and thus cannot use them to generate conscious expectations. The effects can nevertheless be explained by the Interactive Account in terms of automatic top-down influences that combine with bottom-up visual information to determine information processing in vOT.

## vOT selectivity to words and other orthographic stimuli

Several studies have shown activity is higher in response to pseudowords than to words in posterior parts of the occipitotemporal sulcus (*y* = –60 to *y* = –70 in MNI space) and more sensitive to words than to pseudowords in anterior parts of the occipitotemporal sulcus (*y* = –40 to *y* = –50) (for a review, see [40]). However, here we consider the more perplexing pattern of selectivity that occurs at the centre of vOT (*y* = –50 to *y* = –60), where activity has been reported to be greater for: i) pseudowords (e.g. GHOTS) than for consonant letter strings (e.g. GHVST) [41]; (ii) pseudowords than words (e.g. GHOST) [42]; and (iii) low versus high frequency words (GHOST versus GREEN) [43]. This combination of effects cannot be explained by a progressive increase or decrease in vOT response to familiarity (consonants < pseudowords < low frequency words < high frequency words) because responses to pseudowords are higher than those to both unfamiliar consonants and familiar words. Nor can vOT response selectivity be explained by bigram or trigram frequency [44], because greater activation has been reported for pseudowords than for words when bigram and trigram frequency are controlled [42].

The Interactive Account explains vOT responses to different types of stimulus simply, in terms of interactions between bottom-up visual information and top-down predictions (Figure 2). During passive viewing tasks, activation increases for pseudowords relative to consonant letter strings because pseudowords are more word-like and therefore engage top-down predictions from phonological areas. By contrast, activation is greater for pseudowords than for words because, although both activate top-down predictions, there is a greater prediction error for pseudowords. That is, for a previously encountered stimulus (i.e. a word) there is a good match between predictions and the visual representations being predicted, producing minimal prediction error, whereas for unfamiliar pseudowords there is a poor match that increases prediction error and activation in vOT. Similarly, prediction error and activation will be less for high than for low frequency words because high frequency words are more familiar, which means their predictions are more efficient because they call on stronger associations between visual and linguistic codes.

This account also explains apparent word selectivity, such as repetition suppression in vOT for words primed by an identical word but not for those where the prime differs from the target by one letter (e.g. coat–boat) [45]. Clearly, the non-identical prime activates different phonological and semantic patterns than the target word, leading to increased prediction error in vOT [38]. In contrast, small orthographic differences between the prime and the target that result in only minor phonological and semantic changes (e.g. teacher–teach) yield minimal prediction error, resulting in reduced vOT activation [46].

It is important to note that selectivity (in terms of greater activation for one stimulus relative to another) depends on numerous bottom-up and top-down processing demands that change with the task, familiarity with the stimulus, and the degree of overlap between the stimulus and other stimuli that might compete for a response (i.e. the orthographic neighbourhood effect). It is possible that selectivity can be reversed in one context relative to another. For example, during passive viewing conditions, vOT activation can be higher for words than for consonant strings because top-down predictions are activated by words that look familiar. In contrast, in attentionally demanding paradigms (e.g. the one-back task), vOT activation can be higher for consonants than for words [47] because, in the absence of top-down support from semantics and phonology, the visual processing demands of the task are greater for consonants.

## vOT selectivity to words and pictures

When semantic and phonological associations are controlled by comparing written object names to pictures of the same objects, activation in vOT is typically greater for pictures than for written words [48,49], but again, it depends on the combination of the task [10] and the bottom up visual inputs. During a non-linguistic task such as passive viewing, colour decision or a one-back task, vOT activation can be higher for words than for pictures when the physical dimensions of the visual stimuli are matched [2,10], although the location of this effect may be anterior to vOT proper [50]. By contrast, during naming tasks, vOT activation has only been reported as greater for pictures than for words [38,49].

Again, the task-specific reversal of stimulus selectivity can be explained by the Interactive Account in terms of a combination of forward inputs, top-down predictions and the mismatch between them (i.e. the prediction error). Activation related to forward inputs is greater for larger and more complex visual stimuli (e.g. pictures). Activation related to top-down predictions is greater for words than for pictures during non-linguistic tasks because only words have a sufficiently tight relationship with phonology to induce top-down predictions automatically. Activation related to prediction error is higher for pictures than for words during naming tasks because access to phonology is needed to name pictures and words, but the links between vOT and phonological areas are less accurate (more error-prone) for pictures. Thus, the Interactive Account provides a systematic and parsimonious explanation of a previously unexplained range of empirical data.

## Concluding remarks

In summary, we have presented an Interactive Account that is based on a generic framework for understanding brain function [22] (Figure 2). It explains vOT activation in terms of the synthesis of visual inputs carried in the forward connections, top-down predictions conveyed by backward connections, and the mismatch between these bottom-up and top-down inputs.

Although there are many outstanding questions (Box 4), we suggest that: (i) vOT activation to orthographic stimuli increases while individuals are learning to read because inter-regional interactions become established and top-down predictions from phonological and semantic processing areas become available; (ii) vOT activation is greater for pseudowords than for words, and for low relative to high frequency words because of increased prediction error; (iii) greater activation for pictures of objects than for their written names is the combined consequence of more complex visual features, less constrained top-down predictions and therefore increased prediction error; (iv) greater activation for written words than objects is observed when the task does not control for the top-down influence of language on written word processing; (v) damage to vOT impairs reading, object naming and perceptual processing because visual inputs are disconnected from top-down predictions from vOT; and (vi) vOT activation will be lower in developmental dyslexics, in whom top-down predictions from phonological and semantic processing areas are less automatically generated than in age-matched skilled readers.

The automatic interactions between visual, phonological and semantic information that we argue for are a fundamental property of almost all cognitive models of visual word recognition and are necessary to explain a range of reading behaviours [28,31–33]. Incorporating them within a neural framework obviates the need to postulate a novel form of learning-related plasticity (e.g. ‘neuronal recycling’) [7] or reading-specific neuronal responses (e.g. ‘bigram detectors’) [8]. Instead, the Interactive Account relies on well established principles of neocortical function that are not specific to reading, but nonetheless accommodate this recently developed cultural skill.

**Glossary**

**Bottom-up sensory information**: external information arrives at the senses and projects to primary sensory cortices. These drive secondary, tertiary and higher order association cortices via forward connections arising primarily from superficial (layer II and III) pyramidal neurons. Within the ventral occipitotemporal cortex (vOT), the primary source of bottom-up information is visual, presumably from areas V2, V4v, and posterior parts of the lingual and fusiform gyri.
**Generative models**: probabilistic models of how (sensory) data are caused. In machine learning, they include both bottom-up ‘recognition’ connections and top-down ‘predictive’ connections [23]. These models learn multilayer representations by adjusting the top-down connection weights to better predict sensory input. Existing computational models of reading use implicit generative models and share many important features such as interactivity and the use of prediction errors to learn weights (e.g. through back-propagation of errors).
**Predictive coding**: a ubiquitous estimation scheme (developed in engineering) and instantiated in hierarchical generative models of brain function [35,76–78]. Here, cortical regions receive bottom-up input encoding features present in the environment as well as top-down predictions. These predictions attempt to reconcile sensory input with one's internal knowledge of how input is generated. Thus, the function of any region is to integrate these two sources of input dynamically into a coherent, consistent, stable pattern of activity.
**Prediction error**: the difference between bottom-up (sensory) input and top-down predictions. Within vOT, prediction error is minimized when they agree. Any irresolvable mismatch (e.g. when processing pseudowords) elicits prediction error, which elicits an increased BOLD signal response (Figure 2).
**Top-down predictions**: the automatic input a region receives from areas above it in the anatomical hierarchy. These connections attempt to predict the bottom-up inputs based on the context and active features. Important sources of top-down input to vOT are (deep) pyramidal cells in cortical areas that contribute to representing the sound, meaning and actions associated with a given stimulus.

## Box 1 Learning to read and developmental dyslexia

Reading involves linking orthography (i.e. written symbols) to phonology (speech sounds) and semantics (meaning). Learning these associations enhances the ability to predict and perceive the defining visual features of symbols that have been learned. For example, letter combinations will be recognized more efficiently when they are familiar and strongly linked to phonology (e.g. WINE) than when they are less familiar (e.g. WINO) [54]. At the neural level, learning involves experience-dependent synaptic plasticity, which changes connection strengths and the efficiency of perceptual inference.

According to the Interactive Account of ventral occipitotemporal cortex (vOT) function during reading, top-down predictions are conveyed by backward connections from phonological and semantic areas to vOT (Figure 2). These top-down predictions are engaged during the early stages of learning to name objects, and when learning to read words or learning a new orthography. The predictions produce prediction errors, which drive learning to improve prediction. In pre-literates, vOT activation is low because orthographic inputs do not trigger appropriate representations in phonological or semantic areas and therefore there are no top-down influences (stage 1 in Figure 2b). In the early stages of learning to read, vOT activation is high because top-down predictions are engaged imprecisely and it takes longer for the system to suppress prediction errors and identify the word (stage 2 in Figure 2b). In skilled readers, vOT activation declines because learning improves the predictions, which explain prediction error efficiently (stage 3 in Figure 2b). In developmental dyslexics, abnormally low vOT activation [55–60] and reduced functional connectivity between vOT and other language areas [61] are consistent with failure to establish hierarchical connections and access top-down predictions, perhaps because of a paucity of phonological knowledge (i.e. failure to progress from stage 1 to stage 2 [Figure 2b]).

This perspective explains the learning-related increases in vOT activation that have been demonstrated in non-reading pre-school children learning the sounds of letters [62], adults learning sounds and meanings in an artificial orthography [1] and children improving their overt word reading speed [2]. In addition, vOT activation is reduced following visual form learning [1], which demonstrates that learning-related effects are task dependent [1,63]. The Interactive Account explains these effects in terms of experience-dependent plasticity and the resulting increases and decreases in prediction error (Figure 2b). The same learning-related principles apply irrespective of whether the stimuli are letters, words or objects [9,21,64,65].

## Box 2 Damage to the ventral occipitotemporal cortex and pure alexia

Reading impairment is the most notable effect of selective damage to the ventral occipitotemporal cortex (vOT) [3–6]. This deficit is typically referred to as ‘pure alexia’ because speech and language abilities remain intact, as does the ability to write words. Most patients with vOT damage also have difficulty naming objects [66–68], consistent with a generic difficulty linking visual inputs to the language system. Nevertheless, a few patients with vOT damage have been reported with worse reading than naming accuracy [6,69]. This does not mean that vOT is only necessary for reading, because: (i) accurate object naming following vOT damage has only been reported in patients with mild alexia, which manifests in reading speed rather than reading accuracy; (ii) difficulties with object recognition and naming become apparent when the speed of processing is taken into account [6,70]; and (iii) better object naming after vOT damage may be supported by post hoc learning-related changes in other brain regions that provide alternative connections from vision to the language system, with these connections being more successful for object recognition than word recognition.

How does the Interactive Account explain why vOT is more critical for reading than for object recognition? According to the Interactive Account, damage to vOT will disconnect forward and backward connections at all levels of the hierarchy (Figure 2), leading to imprecise perceptual inference. This will have a disproportionate effect on reading because written words comprise the same component parts occurring repeatedly in different, but sometimes highly similar, combinations (e.g. attitude, altitude, aptitude). Object recognition will also be impaired when vOT is disconnected from occipital and higher order language areas, but it will be less impaired than reading when it can proceed on the basis of holistic shape information and a limited number of defining features.

## Box 3 Neuronal properties of the ventral occipitotemporal cortex

Reading-sensitive areas within the ventral occipitotemporal cortex (vOT) lie anterior and lateral to V4 (Figure 1) and correspond most closely to the ventral posterior inferior temporal cortex in non-human primates [71]. Because we are unaware of any single cell neurophysiology data from vOT in humans, we have extrapolated the following three properties of vOT cells from monkey studies. First, individual neurons receive forward afferents from earlier visual fields such as V4 where combinations of simple shapes (forms) such as oriented bars, intersections, angles, arcs and contours are encoded (Figure 1b) [72]. vOT neurons integrate information from these shape elements resulting in complex receptive fields that cannot be characterized solely in terms of example stimuli [73]. Second, vOT neurons tend to have large receptive fields that include the fovea [74]. As a result, they rely on an object-centred reference frame that provides a measure of independence from retinotopic location (Figure 1c). This type of multipart, object-centred receptive field provides a compact, efficient representation that is largely insensitive to the specific placement or size of the stimulus on the retina. Third, each cell contributes to the encoding of multiple visual stimuli; there is no one-to-one mapping between neuronal activity and the orthography of words such as letters, bigrams and trigrams. Instead, encoding a visual word is accomplished via a pattern of firing over a population of vOT neurons. Any given neuron participates in multiple patterns, which can include both written words and other visual stimuli such as objects. Note that the opposite need not be true. Not all vOT neurons will contribute to visual word recognition given the limited set of shapes necessary to encode orthographic forms relative to those necessary for encoding natural scenes [75]. In other words, a neural population response represents a complex stimulus – be it a word or an object – in terms of its constituent elements.

In summary, vOT neurons are general-purpose analyzers of visual forms and underlie all types of complex visual pattern recognition, not just reading. Even the most selective cells respond to various shape patterns, providing a distributed structural code that is highly generative – that is, different combinations of these coding elements can represent a virtually infinite set of visual objects. Visual experience results in plastic changes that tune the receptive fields to facilitate recognition of the most commonly occurring patterns, but this does not alter their fundamental nature; no cells are ‘recycled’ to become reading-specific [7,8]. Consequently, reading relies on the same neurophysiological mechanisms as any other form of higher order vision.

## Box 4 Outstanding questions

• Where are the anatomical sources of top-down phonological and semantic influences and how do they depend on the task and attentional set?
• What are the anatomical pathways linking higher order association cortices to the ventral occipitotemporal cortex (vOT)?
• Are there paths linking vision to language that bypass vOT, and if so, under what circumstances can these sustain reading?
• What are the temporal dynamics of vOT contributions to reading?
• Do the left and right vOT contribute differentially to visual word and object recognition?
• Can direct single cell neurophysiology of the human vOT differentiate between reading-specific neuronal responses and the domain-general neural properties proposed here?
• Would damage (or transcranial magnetic stimulation) to the sources of backward connections to vOT impair the ability to distinguish words, pseudowords and random letter strings?

## References

[1] Xue G. (2006). Language experience shapes fusiform activation when processing a logographic artificial language: an fMRI training study. Neuroimage.

[2] Ben-Shachar M. (2011). The development of cortical sensitivity to visual word forms.. J. Cogn. Neurosci..

[3] Cohen L. (2004). The pathophysiology of letter-by-letter reading. Neuropsychologia.

[4] Leff A.P. (2006). Structural anatomy of pure and hemianopic alexia. J. Neurol. Neurosurg. Psychiatry.

[5] Pflugshaupt T. (2009). About the role of visual field defects in pure alexia. Brain.

[6] Starrfelt R. (2009). Too little, too late: reduced visual span and speed characterize pure alexia. Cereb. Cortex.

[7] Dehaene S., Cohen L. (2007). Cultural recycling of cortical maps. Neuron.

[8] Dehaene S. (2005). The neural code for written words: a proposal. Trends Cogn. Sci..

[9] Song Y. (2010). The role of top-down task context in learning to perceive objects. J. Neurosci..

[10] Starrfelt R., Gerlach C. (2007). The visual what for area: words and pictures in the left fusiform gyrus. Neuroimage.

[11] Xue G. (2010). Facilitating memory for novel characters by reducing neural repetition suppression in the left fusiform cortex. PLoS ONE.

[12] Amedi A. (2001). Visuo-haptic object-related activation in the ventral visual pathway. Nat. Neurosci..

[13] Buchel C. (1998). A multimodal language region in the ventral visual pathway. Nature.

[14] Costantini M. (2011). Haptic perception and body representation in lateral and medial occipito-temporal cortices. Neuropsychologia.

[15] Price C.J., Devlin J.T. (2003). The myth of the visual word form area. Neuroimage.

[16] Xue G., Poldrack R.A. (2007). The neural substrates of visual perceptual learning of words: implications for the visual word form area hypothesis. J. Cogn. Neurosci..

[17] Devlin J.T. (2006). The role of the posterior fusiform gyrus in reading. J. Cogn. Neurosci..

[18] Price C.J., Friston K.J. (2005). Functional ontologies for cognition: the systematic definition of structure and function. Cogn. Neuropsychol..

[19] Woodhead Z.V. (2011). The visual word form system in context. J. Neurosci..

[20] Reinke K. (2008). Functional specificity of the visual word form area: general activation for words and symbols but specific network activation for words. Brain Lang..

[21] Song Y. (2010). Short-term language experience shapes the plasticity of the visual word form area. Brain Res..

[22] Friston K. (2010). The free-energy principle: a unified brain theory?. Nat. Rev. Neurosci..

[23] Hinton G.E. (2007). Learning multiple layers of representation. Trends Cogn. Sci..

[24] Kassuba T. (2011). The left fusiform gyrus hosts trisensory representations of manipulable objects. Neuroimage.

[25] Luders H. (1991). Basal temporal language area. Brain.

[26] Cai Q. (2010). The left ventral occipito-temporal response to words depends on language lateralization but not on visual familiarity. Cereb. Cortex.

[27] Friston K. (2006). A free energy principle for the brain. J. Physiol. Paris.

[28] McClelland J.L., Rumelhart D.E. (1981). An interactive activation model of context effects in letter perception, part I: an account of basic findings. Psychol. Rev..

[29] Seidenberg M.S., McClelland J.L. (1989). A distributed, developmental model of word recognition and naming. Psychol. Rev..

[30] Rueckl J., Seidenberg M.S., Pugh K., McCardle P. (2009). Computational modeling and the neural bases of reading and reading disorders. How Children Learn to Read.

[31] Plaut D.C. (1996). Understanding normal and impaired word reading: computational principles in quasi-regular domains. Psychol. Rev..

[32] Coltheart M. (2001). DRC: a dual route cascaded model of visual word recognition and reading aloud. Psychol. Rev..

[33] Jacobs A.M. (2003). Receiver operating characteristics in the lexical decision task: evidence for a simple signal-detection process simulated by the multiple read-out model. J. Exp. Psychol. Learn. Mem. Cogn..

[34] Hupe J.M. (1998). Cortical feedback improves discrimination between figure and background by V1 V2 and V3 neurons. Nature.

[35] Rao R.P., Ballard D.H. (1999). Predictive coding in the visual cortex: a functional interpretation of some extra-classical receptive-field effects. Nat. Neurosci..

[36] Twomey T. (2011). Top-down modulation of ventral occipito-temporal responses during visual word recognition. Neuroimage.

[37] Yoncheva Y.N. (2010). Auditory selective attention to speech modulates activity in the visual word form area. Cereb. Cortex.

[38] Kherif F. (2011). Automatic top-down processing explains common left occipito-temporal responses to visual words and objects. Cereb. Cortex.

[39] Dehaene S. (2001). Cerebral mechanisms of word masking and unconscious repetition priming. Nat. Neurosci..

[40] Price C.J., Mechelli A. (2005). Reading and reading disturbance. Curr. Opin. Neurobiol..

[41] Price C.J. (1996). Demonstrating the implicit processing of visually presented words and pseudowords. Cereb. Cortex.

[42] Kronbichler M. (2004). The visual word form area and the frequency with which words are encountered: evidence from a parametric fMRI study. Neuroimage.

[43] Graves W.W. (2010). Neural systems for reading aloud: a multiparametric approach. Cereb. Cortex.

[44] Binder J.R. (2006). Tuning of the human left fusiform gyrus to sublexical orthographic structure. Neuroimage.

[45] Glezer L.S. (2009). Evidence for highly selective neuronal tuning to whole words in the “visual word form area”. Neuron.

[46] Devlin J.T. (2004). Morphology and the internal structure of words. Proc. Natl. Acad. Sci. U.S.A..

[47] Wang X. (2011). Left fusiform BOLD responses are inversely related to word-likeness in a one-back task. Neuroimage.

[48] Duncan K.J. (2009). Consistency and variability in functional localisers. Neuroimage.

[49] Wright N.D. (2008). Selective activation around the left occipito-temporal sulcus for words relative to pictures: individual variability or false positives?. Hum. Brain Mapp..

[50] Szwed M. (2011). Specialization for written words over objects in the visual cortex. Neuroimage.

[51] Hegde J., Van Essen D.C. (2007). A comparative study of shape representation in macaque visual areas v2 and v4. Cereb. Cortex.

[52] Connor C.E. (2007). Transformation of shape information in the ventral pathway. Curr. Opin. Neurobiol..

[53] Dehaene S. (2010). How learning to read changes the cortical networks for vision and language. Science.

[54] Goswami U., Ziegler J.C. (2006). A developmental perspective on the neural code for written words. Trends Cogn. Sci..

[55] Blau V. (2010). Deviant processing of letters and speech sounds as proximate cause of reading failure: a functional magnetic resonance imaging study of dyslexic children. Brain.

[56] Brunswick N. (1999). Explicit and implicit processing of words and pseudowords by adult developmental dyslexics: a search for Wernicke's Wortschatz?. Brain.

[57] Richlan F. (2010). A common left occipito-temporal dysfunction in developmental dyslexia and acquired letter-by-letter reading?. PLoS ONE.

[58] Shaywitz B.A. (2002). Disruption of posterior brain systems for reading in children with developmental dyslexia. Biol. Psychiatry.

[59] van der Mark S. (2009). Children with dyslexia lack multiple specializations along the visual word-form (VWF) system. Neuroimage.

[60] Wimmer H. (2010). A dual-route perspective on poor reading in a regular orthography: an fMRI study. Cortex.

[61] van der Mark S. (2011). The left occipitotemporal system in reading: disruption of focal fMRI connectivity to left inferior frontal and inferior parietal language areas in children with dyslexia. Neuroimage.

[62] Brem S. (2010). Brain sensitivity to print emerges when children learn letter-speech sound correspondences. Proc. Natl. Acad. Sci. U.S.A..

[63] James K.H. (2010). Sensori-motor experience leads to changes in visual processing in the developing brain. Dev. Sci..

[64] Turkeltaub P.E. (2008). Development of ventral stream representations for single letters. Ann. N. Y. Acad. Sci..

[65] McCrory E.J. (2005). More than words: a common neural basis for reading and naming deficits in developmental dyslexia?. Brain.

[66] Hillis A.E. (2006). Restoring cerebral blood flow reveals neural regions critical for naming. J. Neurosci..

[67] Hillis A.E. (2005). The roles of the “visual word form area” in reading. Neuroimage.

[68] Marsh E.B., Hillis A.E. (2005). Cognitive and neural mechanisms underlying reading and naming: evidence from letter-by-letter reading and optic aphasia. Neurocase.

[69] Gaillard R. (2006). Direct intracranial FMRI, and lesion evidence for the causal role of left inferotemporal cortex in reading. Neuron.

[70] Starrfelt R. (2010). Visual processing in pure alexia: a case study. Cortex.

[71] Sereno M.I., Tootell R.B. (2005). From monkeys to humans: what do we now know about brain homologies?. Curr. Opin. Neurobiol..

[72] David S.V. (2006). Spectral receptive field properties explain shape selectivity in area V4. J. Neurophysiol..

[73] Brincat S.L., Connor C.E. (2004). Underlying principles of visual shape selectivity in posterior inferotemporal cortex. Nat. Neurosci..

[74] Gross C.G. (1969). Visual receptive fields of neurons in inferotemporal cortex of the monkey. Science.

[75] Changizi M.A. (2006). The structures of letters and symbols throughout human history are selected to match those found in objects in natural scenes. Am. Nat..

[76] Mumford D. (1992). The role of cortico-cortical loops. Biol. Cybern..

[77] Dayan P. (1995). The Helmholtz machine. Neural Comput..

[78] Friston K., Kiebel S. (2009). Predictive coding under the free-energy principle. Philos. Trans. R. Soc. Lond. B: Biol. Sci..

